# Cognitive frontiers: neurotechnology and global internet governance

**DOI:** 10.3389/fdgth.2025.1690489

**Published:** 2025-12-12

**Authors:** Roxana Radu

**Affiliations:** Blavatnik School of Government, University of Oxford, Oxford, United Kingdom

**Keywords:** neurotechnology, internet governance, virtual reality, brain data, privacy, data protection

## Abstract

This article explores the largely uncharted intersection of neurotechnology and Internet governance on the international policy agenda. Neurotechnologies encompass a broad spectrum of functions and applications, from the direct recording or alteration of brain activity to the analysis of emotions and mental states through data collected from wearable devices, applications, and AI-based tools. Innovations such as cochlear implants, sleep optimisation technologies, and immersive educational tools are already available, and significant investments are made in the next generation of devices that blur the lines between mind, machine, and action, posing unprecedented challenges. While some international organisations have begun addressing the ethical and human rights implications of neurotechnology, there remains significant fragmentation and a lack of clarity regarding its integration into Internet governance. Critical issues related to neural infrastructure, standards, access to technologies, and protections for neural data have been overlooked in the 2024 Global Digital Compact and might remain off the agenda for the upcoming 20th review of the World Summit on the Information Society. This contribution underscores the urgent need to analyse the profound implications of neurotechnology, advocating for proactive measures that align with progress made across Internet governance fora, with respect to legal safeguards, multistakeholder consultations and institutional pillars.

## 1 The internet to come: a full-fledged physical-virtual integration

What was once purely speculative—the ability to monitor brain waves, transfer emotions and memories, whether true or false (1), and influence actions in real-time—is now closer than ever. By 2030, a seamlessly interconnected, intelligent, and immersive world built on the next generation Internet (sometimes referred to as Web 4.0) will enable a fusion of digital and physical objects and environments. It will facilitate more advanced human-machine interactions and open new avenues for innovation, growth and sustainable development. From limb rehabilitation to sleep optimisation, these cutting-edge technologies are set to become integral to virtual worlds, spanning domains such as health, education, entertainment, but also the judiciary (2) or the military (3, 4).

Understanding a person's thoughts, preferences, and emotions enables a deeply personalised and responsive experience in virtual worlds, but can also cause unprecedented harm to individuals, communities and societies more broadly. Due to the high sensitivity of the neural processes, neurotechnologies strike at the core of mental self-determination (5) and the very origin of the self. For these reasons, the journey towards immersive world(s) must consider how existing Internet governance frameworks can address neurotechnologies as a key element of the post-2030 agenda. The neurotechnologies available today are able to perform three main functions: 1) brain-imaging (or reading the brain activation patterns); 2) neurostimulation (covering short-term interventions in neural processes); and 3) neuromodulation (which comprises processes inducing longer-term change in brain activity, in particular for medical uses). Advances in the field of neuroscience are also making possible increasingly sophisticated ways to interface directly with neural tissue, opening avenues for both therapeutic and augmentative applications (6).

Experimentation with implantable technologies—such as brain-computer interfaces (BCIs) which link the human brain with computers or external devices (7)—shows promise in supporting bidirectional communication and control, with applications in domains as diverse as telemedicine and gaming (8). Other technical tools designed to perform specific functions (such as cochlear implants for restoring hearing), are increasingly complemented by biosensor technologies that infer sensory, motor, and mental states in a non-invasive way. Commercial devices available today include eye-tracking, video oculography, voice recognition and analysis, sleep movement monitoring, or facial emotion recognition systems. Some wearable devices– such as smart earbuds or XR glasses—-can already monitor neural signals or track physiological responses in real-time (9)

Research on the long-term impacts of commercial neurotech applications is currently lacking (10), as are scientific studies on brain-focused wellness products (11). Neuroenhancement and neuromarketing concerns have only just started to be addressed (12–14). Whether aimed at improving cognitive functions or influencing consumer behaviour, interventions that affect neural processes could cause unpredicted harm to the nervous system and exacerbate individual and societal inequalities, potentially undermining the essence of what it means to be human. Particularly concerning is the fact that these developments take place in a largely unregulated environment, without comprehensive safety and security standards for neurotechnology hardware, software or data in commercial use. Dual-use considerations aside, brain technologies pose new risks to individuals, communities and societies more broadly.

## 2 Current trends in neurotechnology

The field of neurotechnology is advancing quickly, driven by increased specialisation and significant investments from both the public and private sectors, indicating a medium-term market shift. While governments have mainly stimulated neuroscience, with an estimated $6 billion allocated to national brain-research initiatives in the past decade, direct private investments in neurotechnology companies have exceeded $33.2 billion between 2010 and 2020 (15). Consequently, the neurotechnology devices market is expected to grow to $24.2 billion by 2027 (16).

According to a 2023 UNESCO report, the surge in funding has resulted in a 35-fold increase in neuroscience publications and a 20-fold rise in innovations, as measured by patents (15). However, 80% of high-impact publications come from just 10 countries, and six countries are responsible for 87% of neurotechnology patents. The United States alone accounts for nearly half of all global patent applications (47%). Other major contributors are the Republic of Korea (11%), China (10%), Japan (7%), Germany (7%), and France (5%). Together, these six countries hold 87% of neurotechnology patents filed between 2000 and 2020 in the world's five largest Intellectual Property offices (15).

In China and the Republic of Korea, public sector universities and institutions hold a significant number of patents. However, the commercialisation of neurotech is mostly driven by “Big tech” companies in the U.S. Significant investments in the field come from Meta, Neuralink, Alphabet and Kernel (17). The foremost neurotech innovations are primarily patented by multinational tech conglomerates, with IBM (US), Ping An Technology (China), Fujitsu (Japan), Microsoft (US), and Samsung (Republic of Korea) leading the rankings. The analysis of patent clusters shows that key areas of focus include multimodal neuromodulation, seizure prediction, neuromorphic computing, and brain-computer interfaces.

These emerging patent trends show that neurotechnology is spilling beyond clinical settings, paving the way for everyday, non-medical applications. An illustrative example is the consumer-grade electroencephalogram (EEG) headset (18), widely available as meditation, stress measuring or gaming aid. Likewise, major corporations are investing heavily in BCIs that let users operate handheld devices directly with their neural activity (19). On the near horizon, external interfaces for gaming, equipment control and memory and concentration enhancement will continue to be rolled out. While thought-sharing via implanted interfaces may take decades to develop, speech decoders (20) and “typing by the brain” (21) appear much nearer.

The convergence with AI has accelerated neurotechnology advances. The interest in emotion AI (22), in particular, has been on the rise, with applications extending across healthcare, education, marketing, entertainment, and smart home systems. Neuromarketing, as a subfield of advertising that leverages neural data to predict consumer behaviour, has also gained considerable traction. Immersive environments are geared towards a more granular collection of personal data, including brain wave patterns (23). Together, these trends suggest that the groundwork for a post-2030 future is being firmly laid around brain data, promising both groundbreaking innovations and significant societal challenges, as discussed below.

## 3 Internet governance and neurotechnology

Leaping from the lab into the limelight, neurotechnologies raise new questions about ethical concerns, risks and fundamental rights protection. No binding international framework on neurotechnology exists yet: despite the progress made in identifying the lacunae, the mind-technology intersection remains unprotected (24). Until now, the discussion has primarily centered on ethical and legal aspects, with more emphasis on the “neuro” component (25) and less on the technological aspects. Convergence with AI and wearable devices further confounds the governance discussion, straddling different sectors, applications and regulatory mandates.

Less-invasive tools have made neurotechnology safer and more accessible. For medical uses, existing regulatory structures address many core aspects intersecting with health research and clinical practice. Institutional review boards, FDA/EMA-type approval pathways, and emerging voluntary commitments impose safety, efficacy, and informed-consent requirements for devices that record, stimulate, or decode brain activity. Good Clinical Practice standards and post-market surveillance mandates also play an important role in ensuring neurotech can meet the same bar as traditional medical devices.

Despite these foundations, significant gaps remain. Current medical-device regulations were drafted before the proliferation of closed-loop neuromodulators, consumer-grade EEG wearables, and AI-driven neurodiagnostics, leaving unclear how to assess long-term neural plasticity effects, algorithmic bias, or cross-border data-sharing for brain signals (26). A 2025 scoping review of clinical studies involving closed-loop neurotechnologies found that ethical considerations—such as autonomy, data privacy, and equitable access—are rarely addressed in trial protocols, highlighting a disconnect between regulatory compliance and deeper ethical reflection (26). Moreover, there is no universally accepted taxonomy for “neuro-enhancement” vs. therapeutic use, which hampers consistent risk classification (27). Finally, existing oversight lacks coordinated mechanisms for real-time monitoring of off-label applications such as neuromarketing or cognitive-performance boosters, and the legal status of brain-derived data remains ambiguous (28). These gaps are especially problematic when brain data is commercialised, amplifying all of the risks mentioned earlier. Closing these gaps therefore demands more than medical-sector regulation; a comprehensive response will need to combine dedicated neurotechnology frameworks, harmonised international standards, and rigorous scrutiny within broader Internet-governance discussions—an effort that can be supported by existing institutions.

Until now, international discussions on governing neurotechnologies have taken place in silos, disconnected from broader Internet governance debates. In the emerging neurotech field, prominent actors have included intergovernmental organisations like the OECD and UNESCO, professional bodies such as the IEEE, international nonprofits like the Neurorights Foundation and Neuroethics Society, and private companies like Neuralink and Kernel. Wider alliances of policymakers, academics, civil society and citizens are increasingly joining the conversation, as the focus is gradually shifting from responsible innovation towards public governance and oversight of neurotechnologies. This shift offers a timely opportunity to bridge the silos and integrate neurotech into the future Internet and sustainable development agenda.

Significant progress on ethical and legal dimensions has been made since the early 2000s, when ambitious government-funded digital brain research programs first started. The EU Human Brain Project and the US Brain Research Through Advancing Innovative Neurotechnologies (BRAIN) Initiative were launched in 2013, followed by Japan's Brain/MNDS project in 2014, the Korean Brain Initiative in 2016 and the Chinese Brain Science and Brain-inspired Intelligence Project in 2017. Other nation-wide initiatives focused on brain research include the Australian Brain Alliance (since 2016) and the Canadian Brain Research Strategy (2017). More recently, in 2020, the Iniciativa Cerebro Latinoamericana (LATBrain) was established, comprising ten countries from Latin America and the Carribean.

These projects created momentum for articulating key concerns in relation to developing neurotechnologies. These concerns can be understood through three separate lenses: ethical considerations, risk-focused analysis, and rights-oriented perspectives. First, the ethical concerns dimension examines the moral implications of emerging neurotechnologies, questioning how they align with values such as autonomy, beneficence, and justice (5, 29). Second, the risk-based dimension focuses on identifying, quantifying, and managing the concrete hazards—ranging from physical safety and long-term neural plasticity effects to algorithmic bias and data-security vulnerabilities—that these devices may introduce. Finally, the rights-based dimension explores the legal and normative claims individuals can assert over their neural data, including emerging concepts like a “right to mental privacy” and the broader implications for cross-border data sharing and regulatory oversight (30, 31). Together, these three lenses provide a comprehensive framework for analyzing the challenges posed by modern brain-interface technologies.

Because it grants unprecedented access to the inner workings of the human mind, neurotech challenges conventional notions of privacy, autonomy, and dignity: brain-derived data can reveal thoughts, emotions, and health conditions that are often beyond a person's conscious control. The prospect of influencing or augmenting cognition intensifies debates about mental integrity, consent, equitable distribution and social effects. Moreover, the deployment of neural devices in everyday contexts—workplaces, education, and consumer markets—pose multifaced risks, from surveillance to coercion, exploitation, and discrimination. These risks could jeopardise existing fundamental protections such as the right to privacy (ICCPR Art 17), the right to freedom of thought (ICCPR Art 18), the right to non-discrimination (UDHR Arts 2 and 25), disability rights under the CRPD, children's rights under the Convention on the Rights of the Child, or the right to personal security. As the Australian Human Rights Commission notes, the absence of clear legal regimes for brain-derived data amplifies these threats, leaving individuals exposed to surveillance, health or developmental harms, and a loss of agency over their own cognition (23).

As the fields of neuroethics and neurolaw have expanded, advocacy and policy responses have grown at various levels (25). Internationally and regionally, a corpus of soft-law instruments has begun to take shape, while individual states have started enacting formal legislative measures. Since 2020, two specific efforts have gained traction: (1) the development of ethical principles and (2) alignment with human rights.

The first international soft law instrument was adopted in 2019. The OECD Recommendation on Responsible Innovation in Neurotechnology—to which 39 countries have adhered—-outlines 9 principles (see Table 1 below). More recently, in 2023, the Organization of American States has published the Inter-American Declaration of Principles regarding neuroscience, neurotechnologies, and human rights (32), which lists ten principles and builds in transparent governance, oversight and access to effective protection and remedies. In the Council of the European Union, telecommunications and digital ministers signed the first European declaration on neurotechnology in October 2023, committing to a human-centric and rights-oriented approach, while strengthening the EU's competitiveness in the field and its open strategic autonomy in a digital world (33).

**Table 1:** Three sets of commitments issued between 2019 and 2023.

Year	International instruments	Issued by	Commitments
2019	Recommendation on Responsible Innovation in Neurotechnology	OECD	Principles:
			Promoting responsible innovation Prioritising safety assessment Promoting inclusivity Fostering scientific collaboration Enabling societal deliberation Enabling capacity of oversight and advisory bodies Safeguarding personal brain data and other information Promoting cultures of stewardship and trust across the public and private sector Anticipating and monitoring potential unintended use and/or misuse.
2023	Inter-American Declaration of Principles regarding neuroscience, neurotechnologies, and human rights	Organization of American States	Principles:
			identity, autonomy, and privacy of neural activity; protection of human rights in the design of neurotechnologies neural data as sensitive personal data; express and informed consent regarding neural data; equality, non-discrimination, and equal access to neurotechnologies; exclusive therapeutic application respecting cognitive enhancement; neurocognitive integrity; transparent governance of neurotechnologies; supervision and control of neurotechnologies; access to effective protection and remedies for neurotechnology-related issues.
2023	European Declaration on Neurotechnology	Council of the European Union	Call to action:
			Encourage public-private co-operation for the development of rights-oriented, evidence-based and cybersecure neurotechnologies Nurture a dynamic ecosystem that allows to close the gap between research, innovation, and the market Consider accompanying and investment measures in neurotechnologies Facilitate specialised high-level expert discussions Foster dialogue with the European Commission and among Member States Compel European neurotechnology innovators to be aware and adhere to a human-centred and rights-oriented approach by design and by default Actively inform and involve the public Create a trustworthy, transparent, and accountable ecosystem for EU-citizens to use neurotechnology Collaborate with standardisation bodies to explore the need for the creation of standards for neurotechnologies, including cybersecurity standards with a focus on upholding human rights.

In November 2025, UNESCO adopted the first global standard-setting instrument on the ethics of neurotechnology, based on the work of an expert group tasked to create a framework addressing neurotechnology's challenges and benefits. The Recommendation aims to “bring a globally accepted normative instrument that focuses not only on the articulation of values and principles, but also on their practical realisation, through concrete policy recommendations and implementation plans that will be impactful for the global community” (10).

Closely intertwined with ethical approaches, human rights discussions have been divided between updating interpretations of existing rights and creating new ones. Proposals for novel rights aimed at safeguarding the brain, referred to as “neuro-rights” have surged since 2017 (34–36). By now, there is agreement in the philosophical-legal scholarship that “mental privacy, mental integrity, and cognitive liberty […] need to be considered in legal response to advances in neurotechnology” (37). Other proposed neuro-rights include: (1) the right to identity, encompassing control over both physical and mental integrity; (2) the right to agency, ensuring freedom of thought and the ability to choose one's actions; (3) the right to fair access to mental augmentation, ensuring equitable distribution of neurotechnology's benefits for enhancing sensory and cognitive abilities; and (4) the right to protection from algorithmic bias, ensuring technologies remain free from embedded prejudices (38).

This approach has influenced national-level legislation and initiatives, where regulation has been emerging. Chile offers a paradigmatic example, having pioneered a constitutional amendment in 2021 to enshrine neurorights by protecting “cerebral activity and its data” (39), with the Senate also approving a neuroprotection bill at the same time, establishing the rights to personal identity, free will and mental privacy. In August 2023, Chile's Supreme Court upheld these neurorights in a landmark decision by ordering the neurotech company Emotiv to delete all brain data collected from former Senator Guido Girardi. In the United States, the state of Colorado amended the local Privacy Act in 2024 to cover “data generated by the technological processing, measurement, or analysis of an individual's biological, genetic, biochemical, physiological, or neural properties, compositions, or activities or of an individual's body or bodily functions” (40). Despite more attention to legal enshrinment, Magee et al. (31) note that most mental-privacy laws define “neural data” very narrowly, covering only information taken straight from the nervous system (31). They often leave out other kinds of cognitive biometrics—like heart-rate variability, eye-tracking results, or behavior-based patterns—that come from non-neural sources.

Alternative proposals suggest that existing national and international legal systems already safeguard freedoms such as consent, equality, and privacy—concepts that neuro-rights claim to enhance. Internationally, the Council of Europe (41) and UNESCO's International Bioethics Committee (42) have independently determined that rather than creating new human rights, existing human rights laws and regulations should be adapted to address the specific issues posed by neurotechnology. Different civil society groups and human rights experts have issued similar opinions (43, 44).

Around the world, governments have started considering national approaches to neurotechnology governance. Apart from the constitutional reform in Chile, France issued a Charter for the Responsible Development of Neurotechnologies in 2022 (45). Elsewhere, countries like the UK are exploring under what circumstances neural data might be classified as a special category of data under existing data protection frameworks, such as the UK's GDPR (46). Other jurisdictions, such as Spain, have specifically targeted technological convergence. Neurotechnology research is one of the priorities of the Spanish National Strategy for Artificial Intelligence, and a new National Center on Neurotechnologies has recently been launched. The 2021 Charter of Digital Rights (47) included neurorights as part of citizens' rights for the digital era.

The diverse range of international, national, and regional initiatives on neurotechnology present a complex picture. While the specific opportunities and challenges of neurotechnology are well-documented in international discussions, there is less clarity on protections and path(s) forward.

## 4 The intersection of neurotech and internet governance

The ethical, risk-based, and rights-based dimensions of neurotechnology reflect contemporary Internet governance discussions that have evolved through multilateral and multistakeholder discussions since the 1990s and for which institutional structures have been set in place already. Despite this established institutional base, engagement with the current Internet-governance agenda remains sparse, limiting cross-sector dialogue and coordinated policy development. The most striking example comes from the UN Secretary-General's 2021 “Our Common Agenda” report, which identified neurotechnology as a critical frontier issue needing further clarification (48). Subsequently, the UN Human Rights Council has requested a report on neurotechnologies and their potential effects on human rights, whose final version was presented to the Council in March 2025 (49). Ahead of the UN *Summit of the Future*, however, the work stream on the Global Digital Compact and its agreed version (50) have not included a single reference to neurotechnology, despite wider engagement with emerging technologies in the text (51).

The incorporation of neurotechnologies into the digital ecosystem introduces novel variables for Internet-governance deliberations—most notably the need to accommodate ultra-low-latency communication protocols, secure end-to-end encryption for brain-derived data streams, and interoperable standards that allow heterogeneous devices to communicate without compromising safety or privacy. Yet the discipline of Internet governance brings a mature analytical toolkit that is directly applicable to these challenges. Decades of scrutiny concerning the distribution of power among state actors, private corporations, civil society, and technical communities have yielded robust multistakeholder processes, normative frameworks, and institutional mechanisms, from technical bodies to the UN-led Internet Governance Forum (52–55). These structures provide a strong basis to mediate the complex policy questions now arising around neurotechnology, such as who should define technical specifications for neural-data transmission, how equitable access to neuro-digital infrastructure can be ensured across socioeconomic and geographic boundaries, and what accountability regimes are appropriate for entities that collect, aggregate, or repurpose neural data for commercial or governmental purposes. Moreover, the convergence of neurotechnology with immersive virtual environments—augmented reality, virtual reality, and mixed-reality platforms—magnifies the urgency of synchronising governance efforts.

At the nexus of Internet governance and emerging neurotechnologies, two primary axes of convergence emerge: (1) infrastructure, standards, and access—-the technical backbone that supports the routing, storage, and computation of neural-signal traffic, with its access and control points; and (2)** **neural data—the raw and brain-derived information captured by brain-computer interfaces, closed-loop neuromodulators, and consumer-grade wearables. Both are discussed in more detail below.

### 4.1 Infrastructure, standards and access

In the Web 4.0, many neurotechnologies are expected to operate as part of complex wireless ecosystems, which can combine unimpeded movement with higher levels of network connectivity to allow for faster processing of neural data. The underlying infrastructure might be part of the global internet (operating on existing networks) or distinct and specific for virtual worlds. Significant investments from private companies have targeted the creation of “enabling” platforms (56) for neurodevices, equivalent to operating systems for computers or mobile phones. The dual-use nature of neurotechnologies could also incentivise the separation of infrastructure or the unilateral establishment of rules, which is why it is primordial to involve technologists, clinicians, ethicists, regulators, and end-users in nascent policy discussions.

In closed-loop neuromodulation and BCI control loops, ultra-low-delay communication pathways are needed. In more generic uses, the heterogeneity of devices—from implants to consumer-grade wearables—necessitates interoperable protocol stacks and standardised data schemas that can be universally interpreted across manufacturers and jurisdictions. In the absence of interoperability standards and cybersecurity protocols, neurotechnologies pose important challenges to the Internet governance ecosystem. The evolution of the Internet has already been hindered by unequal participation in technology design and security issues (54). Therefore, international discussions on neurotechnology need to consider the value of non-proprietary solutions and standardisation from the outset. These technologies have global consequences, making it essential for international standardisation efforts to be participatory and inclusive of perspectives from various groups of stakeholders around the globe.

Access issues are equally important. In an already uneven landscape with R&D concentrated in a few geographic regions and driven by technologically-advanced countries, the distribution of benefits comes into question. Adding to it, the significant concentration of AI computing power and manufacturing resources in the hands of a few tech giants already structure the market for neurotech. Geopolitical interests and trade wars can also affect the development of the infrastructure, limiting the choices available to middle-income and lower-income countries and regions. Access to technological and medical infrastructure is especially critical in the least developed countries and small island developing states, where communities experience significant digital divides. It is crucial to ensure equal access to the infrastructure and to the latest scientific and technological knowledge.

The standardisation of neurotechnologies is uneven and incomplete, and their integration within the broader Internet systems is not adequately covered by current efforts. While safety, security and privacy are recognised as top priorities, there are sigificant limitations to addressing these issues using standardisation. These include a lack of agreed-upon terminology, limited community engagement, insufficient standards for data sharing, limited reporting on neurotech developments, and the need to specify complementary standards that scale from consumer to clinical applications (57). Alignment with existing frameworks on emerging technologies is also much needed in order to address wider societal and environmental implications. This would ensure safe implementation techniques, adequate security layers and trust in the technology (58). This approach should also integrate product lifecycle and disposal considerations from the outset. Although non-for-profit and professional organisations have started to advocate for a “Technocratic Oath” (59) or an “ethical-by-design” approach (60), standard-setting organisations—including Internet-related ones—are not consistently involved in testing and securing the networks reaching the brain.

Cutting across infrastructure, standards and access issues, sustainability considerations need to be integrated into governance frameworks from the start. Neurotech uses critical materials and device components resulting from processes that are detrimental to the environment and create toxic waste (42). Standards for environmental sustainability introduced early can help reduce their harmful impact, as various international commitments point to (10, 61). As the science around technological convergence and biodegrability progresses, there is an opportunity to leverage technological leapfrogging alongside sustainable growth, particularly in resource-constrained settings. In the near future, changes in the business models are also needed, shifting the start-up focus on quick gains and short-term objectives towards longer-term vision and plans required for neural interface work (17).

### 4.2 Neural data and adequate protections

UNESCO's 2024 draft Recommendation on the Ethics of Neurotechnology distinguishes between neural and cognitive biometric data, but treats them in tandem as “uniquely sensitive because they provide deep insights into the pre-behavioural processes that underpin our mental states and cognitive functions” (10). While the former refers to the brain activity patterns, thought and memory, cognitive biometric data comprises all other inferences about mental states (often AI-enabled) derived from devices and biosensors. Both types of data can influence neural processes in ways that are unanticipated or undesirable, affecting individual mental states and potentially transforming their societal interactions. Ensuring discussions around a “special category of data” protections have been interlinked with privacy risks inherent in capturing, storing, and processing neural signals, regardless of whether they originate from medical devices or consumer-grade wearables. Similarly, equitable access to (longitudinal) neurodata could be complicated by data sharing practices (61), particularly when it comes to cultural differences and the diversity of governance systems around the world.

Debating privacy has been at the core of internet governance debates for at least two decades (62–64), evolving from voluntary commitments to a legal *acquis* in the form of the EU General Data Protection Regulation (GDPR) or the California Consumer Privacy Act (CCPA), in force since 2018 and 2020, respectively. These regulations provide baseline privacy and data security protections, mandating consent, data minimisation, and purpose limitation, and codifying principles such as privacy-by-design and encryption. Yet, such legal frameworks are typically broad and flexible and do not account for unintended uses of neural and cognitive biometric data. Recent reports (65) and academic studies (31) show that all the major firms in BCI, extended reality, and fitness-wearable sectors routinely gather cognitive biometric data and claim broad rights to retain the neural data they collect, often under vague or permissive policies.

The concerns about lax industry practices around the collection, storage, use, and sale of cognitive biometric data are amplified by the fact that many privacy regimes treat de-identified or aggregated data as non-personal, allowing companies to sidestep consent requirements. While some firms claim to share only “aggregate” data for vaguely described (research) purposes, others publicly assert that they do not sell users' personal data, but reserve the right to leverage the same data (including de-identified or aggregated sets) for targeted advertising. However, neural and cognitive biometric data are more difficult to anonymise, increasing the potential for identification (66) beyond the initial context of data collection. From a cybersecurity perspective, misuse might include “hacking the brain” (67) to cause disruptions, as well as new forms of data tampering and user impersonation in virtual worlds.

Related security and surveillance concerns include questions about who collects the data, for how long, where they are stored and local regulations regarding companies' obligations to share data with the host country. Vulnerable populations, as well as those in vulnerable (mental) states, require additional safeguards. For example, the UN Committee on the Rights of the Child argued that practices relying on “neuromarketing, emotional analytics, immersive advertising and advertising in virtual and augmented reality environments to promote products, applications and services should [also] be prohibited from engagement directly or indirectly with children” (68). In this regard, the brain's development and its plasticity require special protections, especially considering the potential to influence life chances and future opportunities. Thus the life cycle approach to neural data and related technology is all the more important.

Consequently, there have been two main proposals for addressing neural data: carving out a separate legal right to mental privacy (as discussed above) or integrating adequate protections within existing data-protection regimes. The digital rights agenda in Internet governance serves as a cautionary tale. Despite being on the international agenda for over a decade, its interpretation is evolving with emerging technologies, leading to very different levels of protection across the globe. For Susser and Cabrera (69), neurotechnologies raise novel privacy stakes, comparable to those posed by other advanced data-collection tools in genomic sequencing or online surveillance) and are best addressed as part of existing legislation by adding context-appropriate safeguards, transparency, and accountability. Lessons from Internet governance show robust oversight takes decades to build and cannot be achieved by self-regulation alone (70, 71). Some of the existing mechanisms for governing the Internet—such as standard-setting bodies that codify protections, transparency watchdogs and multi-stakeholder forums that feed into policymaking could support the development of a more coherent, globally interoperable approach to neurotechnology safeguards.

## 5 The opportunity to govern neurotechnologies

The nascent national and regional frameworks addressing neurotechnology, whether directly or indirectly, remain fragmented and incomplete efforts, with many regulatory and protection gaps. As such, they are likely to fall short in addressing the critical challenges outlined in this review, specifically those related to (1) infrastructure, standards, and access, and (2) neural data. Crafting new international treaties to address these concerns is notoriously challenging, which makes convergence with the Internet governance agenda essential. Efforts by individual countries to regulate emerging technologies are often insufficient, raising concerns that neurotech companies might operate from or relocate to more favourable jurisdictions. On the other hand, soft law instruments lack formal enforcement mechanisms, rely on voluntary compliance and often result in inconsistent applications. For a domain as critical as neurotechnology, effective governance needs to go beyond to reflect societal concerns about the broader digital transformation.

Previously siloed efforts are beginning to come together as a result of technological convergence, in particular around AI and wearable technology. At the regional level, the EU AI Act outlines different levels of risks induced by the development and deployment of AI systems “with the objective to or the effect of materially distorting human behaviour” (72)*.* While regulatory discussions in the context of emerging technologies might incorporate neurotechnology, they are not sufficiently advanced to capture the unique challenges posed by it. These new challenges, likely to affect the very essence of being human, require thorough discussions across all Internet governance and policy venues, as well as in specialised virtual worlds and Web 4.0 forums. Before definitions of key issues are agreed and governance trajectories are locked in, there is a need to include a more diverse group of actors in this process, including the different Internet governance communities active for decades. As a first step, the roles and responsibilities of the state and of regional and international organisations need to be clarified in relation to a domain that is private-sector led.

Broadly formulated principles for the development of neurotechnology help in building consensus across borders, but remain insufficient without governance instruments with binding power. Existing internet governance frameworks and processes for a trusted Internet continue to have neurotech as their blind spot. The Global Digital Compact is a case in point, despite referencing emerging technologies generically. Similarly, the preparatory work for the World Summit on Information Society 20th review (known as the WSIS+20) has not specifically designated cutting-edge technologies that focus on the brain. Increasing awareness, transparency and safety standards for neurotechnologies used for non-medical purposes is a must for a stable and equitable Internet future. Updating and tracking neurotechnology should become a routine part of the Web 4.0 policy conversation, with coordinated efforts across international venues to broaden current institutional mandates. This would allow future Internet-governance work to place a dedicated emphasis on protecting neural data, pairing technological progress with safeguards for the brain—the most sensitive part of the human body.

## 6 Conclusion and way forward

Neurotechnologies are still in their early stages of development, but their potential is immense, especially when paired with AI. By leveraging neural data, these innovations promise to transform both medical and non-medical applications, fundamentally altering our daily lives in ways we have yet to fully grasp. As they develop, they raise urgent questions about access to the underlying infrastructure and technical standards, as well as neural data safeguards for both individual users and communities more broadly.

Nearly all of today's transformative neurotechnologies are connected to the Internet. Yet most international and national discussions often occur in isolation from broader Internet governance conversations. This disconnect ignores the need for a unified approach that integrates neurotechnology into existing frameworks. This review examined the importance of addressing these concerns alongside Internet policy and emphasised the urgency of taking action sooner rather than later. To address these pressing challenges, a collective effort is essential—one that invites diverse stakeholder input into the development and regulation of these technologies as part of ongoing Internet governance initiatives, using the post-2030 agenda as a stepping stone.

In the short term, the deployment of neurotechnology for non-medical purposes should be top of the agenda for Internet policy-makers. This technology adds complexity to existing digital infrastructure and regulation dynamics, accelerating the need to implement clear safety and security standards across the stack. As the post-2030 sustainable development agenda is redefined, it is time to consider how neurotechnology can fit into future-oriented frameworks under negotiation, including the WSIS+20 review due in December 2025.

In the long-term, neurotechnologies could reshape human experience and societal structures in ways we are only beginning to imagine. Failing to address these concerns promptly could deepen the existing disparities and vulnerabilities and create new ones, impacting both users and the wider Internet ecosystem. Equitable access to digital and neural infrastructure, coupled with robust governance mechanisms, will be crucial for a future where neurotechnology is woven into the fabric of our lives.
